# Amendment of Medical Benefit Regulations

**Published:** 1913-05-10

**Authors:** 


OUR
### NATIONAL INSURANCE ACT DEPARTMENT.

### LII.—Amendment of Medical Benefit Regulations.

THE regulations of the Insurance Commissioners under which medical benefit was administered during the first quarter after the benefit came into operation have already undergone considerable modification. It will be remembered that the first agreements entered into by doctors who undertook service on the panels were of a provisional character. These agreements expired on April 14, and their place has been taken by a much simpler document. The principal modification, so far as the doctors are concerned, lies in the simplification of the records that have to be kept. Instead of the cumbersome register, a card system of record has been adopted, and the triplication of entries has been avoided.

The cards are drawn up so that they show on the reverse side the surname, Christian names or initials, and the address of the insured person. When the cards are sorted into alphabetical order, reference to any card that may be required is, from the particulars just mentioned, a very simple matter. On the obverse of the card spaces are provided for recording the attendances, and although the cards measure no more than 6 inches by 5 inches they suffice to allow of the records for a full calendar year, with a space allotted for each day. At the foot of the obverse side space is provided for making brief quarterly summaries, giving the nature of the illnesses, and the number of attendances during the quarter.

This is a great improvement upon the old system of detailed records in registers, though there is the corresponding disadvantage that, as the cards last for one year only, there will be the necessity for rewriting the essential particulars on the new cards before the old ones are returned to the Insurance Committee at the end of the year.

### MEDICAL BENEFIT AND TEMPORARY RESIDENTS.

The alteration in the method of keeping records is, as we have already said, the most important modification of the regulations from the panel doctors' point of view. There is, however, a further alteration which is, perhaps, of even greater importance from the points of view of the insured persons and of the officials of the Insurance Committees. This alteration is described in an official memorandum, numbered 161 I.C., and deals with the question of medical benefit for insured persons who change their residence for a short period or for a temporary purpose only.

The original regulations required insured persons to give notice to the Insurance Committee for the area in which they reside, whenever they remove, and provided for a transfer of funds from the old Committee to the new in proportion to the period for which the insured person remains in the area of the new Committee. This regulation, apparently, appeared to its framers to be quite sound, but it overlooked the fact that the British public are not yet abjectly educated in officialdom, and that, in consequence, as the memorandum naïvely remarks, "experience has shown that insured persons cannot be induced to give notice unless they are ill, or expect to be ill at the time of removal." Seeing, then, that the financial provision for medical benefit is 9s. per annum for each insured person, whether ill or well, it necessarily follows that the result of the omission of the healthy to give notice of removal is that the transfer of funds from one Committee to another on account of those who are ill is inadequate to defray the cost of their treatment.

### THE NEW SYSTEM.

To overcome this difficulty the Insurance Commissioners convened a conference of medical men from the areas mainly affected, and the matter was also discussed at a full meeting of the joint Advisory Committee. The new scheme was based on the suggestions made at these meetings, and in view of the expert character of the advice it is hoped that the scheme will meet all requirements and enable satisfactory arrangements to be made for medical treatment of certain classes of insured persons for whom, under the old capitation scheme, this had been difficult.

Commencing with the present quarter—that is, from April 15—the treatment of all temporary residents by doctors on the panel is being paid for on an attendance basis and not on the capitation system.

The following is the scale that has been adopted for calculating the remuneration:

| | £ | s. | d. |
|---|---|---|---|
| Attendance on the patient at the practitioner's residence, surgery, or dispensary ... ... | 0 | 2 | 0 |
| Visit to patient's residence ... ... ... ... | 0 | 2 | 6 |
| Special visit—that is, a visit paid by the patient's desire on the same day as a call received after 10 A.M., or on Sunday ... ... ... ... | 0 | 3 | 6 |
| Night visit—that is, a visit made between the hours of 8 P.M. and 8 A.M. in response to a call received between those hours ... ... ... | 0 | 5 | 0 |
| Surgical operation requiring local or general anæsthetic, or case of abortion or miscarriage | 1 | 1 | 0 |
| Administration of general anæsthetic ... ... | 1 | 1 | 0 |
| Setting of fracture ... ... ... ... ... | 0 | 10 | 6 |
| Reduction of dislocation ... ... ... ... | 0 | 10 | 6 |

This scale is subject to revision by the Insurance Commissioners in consultation with the local Medical Committees.

### AN INDIRECT ADVANTAGE.

The fees mentioned do not necessarily represent the actual amounts which the doctors will obtain for the services they render, but the actual payments will be in proportion to the scale. It is, of course, impossible to say whether the sum at the disposal of the Insurance Commissioners in the central fund to which contributions are to be made on the basis of 9s. per annum for all insured persons, whether ill or well, will be sufficient to meet the payment of the fees in full.

If after the payment for drugs has been made out of the central fund the sum in hand is sufficient to pay the fees in full according to the scale this will be done. If, however, for example, the fund in hand is only sufficient to enable the Commissioners to pay at the rate of 15s. in the £, then for each attendance at his surgery to a "temporary resident" the doctor will receive 1s. 6d. or 15s. 9d. for a surgical operation requiring an anæsthetic. It will thus be seen that there will be an indirect advantage in the system, because it will make it possible to institute a comparison between the scale of fees and the sum allowed for attendance on the capitation basis. It will probably be agreed that the scale of fees is reasonable for the class of patients who come within the scope of the National Insurance Act, and thus there will be a very ready means of testing the adequacy of the present capitation allowance of 9s.

### The Central Fund.

We have referred to the central fund, and must now explain the plan upon which this is constituted. An investigation is to made at the end of each year to determine the "case value" for persons medically treated within each Committee area. Thus, if it be found in a particular Committee area that one insured person out of every three has on the average received medical treatment during the year, the "case value" will be 9s. × 3, that is £1 7s. In other words, the average cost of medical benefit for those members who have actually been ill will be £1 7s. It is this sum, whatever it may be, which is to be taken into account in calculating the transfer of funds from the Insurance Committees to the central fund. By this means each Insurance Committee will pay neither more nor less than the normal average sum for those insured persons residing within its area who, while temporarily resident elsewhere, require medical treatment. A person is not to be deemed to be temporarily resident elsewhere than in the area in which he permanently resides for more than three months. If the residence continues for so long a period as three months he must at the end of that time be transferred to the new Committee, and the doctor attending him will be paid thereafter on the normal capitation plan.

### How Insured Persons are Affected.

The chief advantage of the new system, in our view, is that it enables insured persons who are temporarily away from home to obtain medical treatment as part of the medical benefit provided by the Act. Under the former arrangement it was practically impossible to obtain the services of a doctor, because the terms of remuneration when only sick persons came to him were so entirely inadequate. The new system tries to meet such objection, and consequently insured persons should not experience the difficulty that many have encountered during the first quarter in obtaining treatment. This applies in particular to commercial travellers, members of travelling theatrical companies, and the like, who by reason of their occupation do not for long remain in the same place. The memorandum states that "some evidence will obviously be required of a temporary resident before he can obtain medical benefit outside his own Committee's area to the effect that he is an insured person, entitled to medical benefit, and that he is on the register of the Committee from whose area he comes. . . . For this purpose a green voucher has been prepared to be issued to the applicant by the Committee for his usual place of residence. . . . On the insured person presenting himself for treatment the doctor will retain the voucher, and enter upon it from time to time particulars of the services rendered . . . and at the conclusion of the treatment the doctor will forward the voucher to his own Committee."

The procedure is, therefore, quite simple, and the only difficulty we can foresee is in the case of a person who before leaving home does not obtain a green voucher. It is conceivable that there may be considerable difficulty in obtaining the voucher through the post, and there must of necessity be some delay. However this may be, we welcome the new system as a great advance on that of its predecessor. This is one of the ways in which experience has already shown the way to improvement, and we look confidently for the same evidence in the amending Bill, the introduction of which is promised at an early date.

